# Book Notices

**Published:** 1882-11-20

**Authors:** 


### *BOOK NOTICES.*

THE PROBLEM OF HUMAN LIFE—Embracing the "Evolution of Sound" and "Evolution Evolved," with a review of the six great modern scientists, Darwin, Huxley, Tyndall, Haeckel, Helmholtz and Mayer—thirty-fourth thousand. By A. Wilford Hall, New York. Hall & Co., 23 Park Row. Price $1.00.

We have examined the above work with more than usual interest, and must acknowledge ourself constrained by force of argument to yield some of our preconceived opinions on scientific subjects. To be properly understood or appreciated, the work must be carefully studied, and this we have not done, having been carried hurriedly through it by the novelty and interest of the writer's views.

The conclusion seems inevitable that the wave theory of sound so long accepted as true by the scientific world is in reality a falacy, having no foundation in fact. This we think is clearly established by the writer in this book.

The position taken by the author that life in man and all living creatures is a substantial entity, consisting of real, though incorporeal substance, is exceedingly plausible. He holds that everything, indeed, is substance, and that the difference between material and immaterial objects is only in degree of density or attenuation. Electricity, light, heat, magnetism and the spirit of man—even God himself—he regards as substantial entities. To appreciate the strength of his argument in favor of this theory one must read the work. We pretend not here to explain his views, and only refer in general terms to the work as being one of rare interest and ability, worthy the perusal and study of intelligent and thinking men. His criticisms upon the doctrines of the evolutionists are very forcible and interesting, and constitute the only satisfactory rebuttal that has yet appeared to the doctrines of Haeckel, Darwin and other materialistic writers.

A PRACTICAL LABORATORY COURSE IN MEDICAL CHEMISTRY. By John C. Draper, M. D., Professor of Chemistry in the Medical Department of the University of New York, and of Physiology and Natural History in the College of the City of New York. William Wood & Co., 56 LaFayette Place, New York, 1882. McGarrity & Laird, Agents, Atlanta, Ga.

This is a snug little work, containing an abridgment or condensed outline of Prof. Draper's Laboratory Course, and admirably arranged to give to the student a practical knowledge of those Chemical manipulations and tests which every intelligent physician ought to understand and is expected to possess. These are important in diagnosis, in questions of Medical Jurisprudence and in many hygienic problems which are likely to arise in the experience of every practitioner. The book is arranged with alternate blank pages for the convenience of the student in taking notes, etc. The alphabetical list of Symbols and Formulæ will be found exceedingly instructive and useful.

QUIZ COMPENDS, No. 1.—QUESTIONS ON ANATOMY. By Samuel O. L. Potter, M. A., M. D., Author of Index of Comparative Therapeutics, and of the Lea Prize Essay of the Jefferson Medical College on Dyslalia; A Study of Speech and its Defects, with sixty-three illustrations. Philadelphia: P. Blakiston & Son, 1012 Walnut street, 1882.

A book of Questions and Answers in Anatomy, very convenient and useful to the teacher of Anatomy and to the medical student. Superfluities are omitted and the cream and pith of the essential portions of the Science only included.

ON ASTHMA, ITS PATHOLOGY AND TREATMENT. By Henry Hyde Salter, M. D., F. R. S., Fellow of the Royal College of Physicians, Physician to Charing Cross Hospital and Lecturer on the Principles and Practice of Medicine at the Charing Cross Hospital Medical School. First American from the last English edition. New York: Wm. Wood & Co. McGarrity & Laird, Agents, Atlanta, Ga.

The work is illustrated, contains 279 octavo pages, is well written and we may say is exhaustive of the important subject of which it treats. In treating hay fever the continued use of strychnine during the attack is mentioned as an absolute cure with many persons. A cruise on a yacht is also mentioned as an absolute specific, because it removes the patient from the cause of suffering. To relieve the paroxysms of Asthma stimulants in the shape of strong coffee and whisky are favorably mentioned.

AN ESSAY ON THE EVILS AND USES OF TOBACCO. By Rev. I. L. Kephart, A. M. Written in competition for a prize of fifty dollars offered by Rev. W, S. Titus, of Charlotte, Mich. Dayton, Ohio, United Brethren Publishing House, 1882.

A little work of seventy-five pages, well written, and containing useful and instructive facts on the great evil of the use of tobacco.

TRANSACTIONS of the Medical Society of the State of West Virginia—14th and 15th Annual Session, 1881–'82, held at Wheeling, May 24, 1882—two volumes in one, making a work of 840 octavo pages.

The addresses and papers are, many of them, able and interesting. We have not, at present, space to mention them as they deserve.

The officers for the present year are as follows:

*President*—Dr. B. W Allen. *Vice-Presidents*—Drs. W. L. Grant, J. H. Manown, C. Shriver. *Secretary*—Dr. S. L. Jepson.

